# ‘Other Spaces’ for the *Dangerous Dead* of Provincial England, *c*.1752–1832

**DOI:** 10.1111/1468-229X.12534

**Published:** 2018-01-23

**Authors:** ELIZABETH T. HURREN

**Affiliations:** ^1^ University of Leicester

## Abstract

The Murder Act (1752) decreed that homicide perpetrators should be hanged and sent for post‐execution punishment. This article explores the event management of criminal dissections by penal surgeons *in situ*. It reveals that the punishment parade of the condemned did not stop at the scaffold, contrary to the impression in many standard historical accounts. Instead, ordinary people accompanied criminal corpses to many different types of dissection venues. Penal surgeons hand‐picked these performance spaces that were socially produced for legal and practical reasons. They had to be able to process large numbers of people who wanted to be part of the consumption of post‐mortem ‘harm’ in English communities. Event management on location had to have emotional and visual appeal, moral coherence, be timed appropriately, and, if successful, would enhance the deterrence value of the capital code. Yet, managing the ‘dangerous dead’ involved a great deal of discretionary justice with unpredictable outcomes. It often happened in ‘counter‐sites’ of punishment in the community and involved a great deal of immersive theatre. Some events worked well, others threatened the social order. In ‘Other Spaces’ the ‘Dangerous Dead’ was hence a fascinating feature of the Murder Act outside the Metropolis from 1752 to 1832.

## I

The Murder Act [25, George II, c. 37, 1752] is central to criminal histories of the long eighteenth century.^1^ It stipulated that homicide perpetrators were to be ‘*hanged by the neck until dead*’ on a public gallows. That death penalty reflected the Biblical commandment ‘Thou shall not kill’ and *Lex Talionis*, the English common law of retaliation.^2^ Executions consequently attracted enormous crowds that congregated at hanging trees across England. In crime studies these public spectacles have often been written‐up exclusively,^3^ even though judges also ordered either a dissection by a penal surgeon, or the criminal corpse was hung in chains on a gibbet.^4^ Recently a consortium of scholars has looked with renewed historical efforts at the activities of medico‐legal officials who managed the entire punishment journey of the condemned, known in popular culture as the *dangerous dead*.^5^ New evidence has uncovered sustained public interest in post‐execution punishment events staged in prominent penal spaces in the community. This article builds on these revisionist perspectives by broadening the narrow anatomical focus of most work written about criminal dissections covering the Murder Act from 1752 until it was repealed by the Anatomy Act in 1832.^6^

New research identifies the types of venues requisitioned for post‐execution events in provincial life. It analyses how exactly dissection premises were event‐managed by medico‐legal officials on duty. Archival material illustrates the spatial architectural arrangements and what these reveal about the theatrical post‐mortem display. Taken together, it will be shown that criminal dissections personified on a much broader cultural canvas ‘the social meanings attached to the body which could vary widely according to culture, and period’.^7^ For, as we shall see, large post‐execution crowds often followed the condemned cut down from the hangman's rope. They did so, to be seen, and be part of, the capital justice that exemplified post‐mortem ‘harm’ in familiar public social spaces throughout England.

Cutting up the criminal corpse was far more complex than standard histories of the capital code have tended to convey for England.^8^ It required a lot of coordination by medico‐legal officials under the Murder Act. In practical terms, there was a working‐choreography that typically involved, first, an *autopsy* by a surgeon, tasked with opening up the criminal corpse and examining the major organs in the chest cavity to make sure the condemned had expired on the gallows.^9^ In surgical parlance this medical checking‐mechanism was dubbed *anatomisation*: an old anatomical term revitalised for the capital code. This was then followed by a second penal procedure called *dissection to the extremities*: little human material was left to bury. It followed that each penal step entailed different types of audience participation. Event management in the English localities was thus dissimilar to that at Surgeons’ Hall in London whose iconic status has obscured the regional settings and timings of provincial punishment rites. Representative examples in this article thus illustrate the temporal and spatial settings in which the public typically viewed the corpse, and how the scrutinizing of the executed body was an active process for the assembled spectators involved in the display, event management and performance of capital justice outside the metropolis.

To avoid overlap with this author's previous published work, extensive new record linkage work has been undertaken exclusively for this article. Source material is drawn from Sherriff's Cravings at the National Archives, Assize records of provincial court cases, newspaper reporting and medical accounts of anatomical events. In unison these trace the intersecting, sometimes clashing, temporal dimensions of punishing the corpse by dissection. All took place in a wide variety of public settings with theatrical potential in the community. Each had their own specific timings, in which penalties were staged for practical, as well as legal, reasons. Events thus attracted diverse spectators with judicial, theological and public apprehensions of punishment. Even so, in all cases on a national basis, the latest research has established that 80 per cent of those convicted of murder were sentenced to a criminal dissection from 1752 until 1832 (as in Table 1). It is time therefore to look beyond the gallows in a history of punishment.

**Table 1:** Sentencing outcomes, homicide convictions: the Murder Act in England, 1752–1832

Sentencing outcome(s)	Number(s)	Percentage %
Bodies made available for dissection	923	80%
Bodies hung in chains	144	12%
People pardoned	97	8%
Miscellaneous (suicides)	2	<1%
**National total: post‐mortem punishment**	1166	100%

*Sources*: The National Archives, Sheriff's Cravings for England, 1752–1832, cross‐checked to Assizes Records, County Record Office(s).

Section II thus begins by outlining how dissected bodies were understood in more‐than‐anatomical terms during the Enlightenment in English society. In terms of the Murder Act's application, it is important to set in context why it was that post‐execution punishment events appeared to personify a more publicly situated sense of ‘display’ that became familiar to, and was expected by, crowds that attended. Likewise new source material generated needs to be situated in the social production of performance spaces, with their inherent sense of theatricality embodying dissection practices. Of relevance are the social theories of Henry Lefebvre, Michael Foucault and David Wiles, who together have encouraged scholars to explore neglected ‘micro‐narratives’ of performance spaces.^10^ Following their example, it is feasible to relocate the physical relationship of performer (corpse), spectator (assembly) and environment (venue) in capital crimes that underscored a sense of community and belonging. The advantage of adopting this approach is that detailed record linkage work can begin to engage with the sorts of visual communication that once took place inside punishment spaces that were all about deterrence, display and dissection. As Wiles argues, this scholarly endeavour ‘is not a *history about the past* as such’, but rather it seeks to rediscover historical prisms in the archives that illustrate the ways there are of ‘creating meaning from the scattered debris’ of ‘*what once was*’.^11^

Having assembled significant new source material, this article can pinpoint in Section III the spectrum of dissection premises requisitioned. For, as we shall see, in provincial life well‐organized event management was crucial for the effective display of the criminal corpse condemned to post‐mortem ‘harm’. Seldom therefore were the places designated for a criminal dissection an arbitrary choice in the community because each location had to determine the deterrence value of the capital sentence in an English locality. Even so, it would be misleading to assume that the attending crowds did not have agency in this public occupation with capital justice. Indeed, Matthew White has recently argued that crime histories have been too inward‐looking because they have tended to ‘aggregate’ the so‐called ‘mob’ into a ‘faceless crowd’ under the Murder Act.^12^ Instead of presenting ‘depictions of … avid execution‐going’ – which he argues have tended to be ‘consistently two‐dimensional and frequently impressionistic’ – more scholarly attention needs to be devoted to the ‘social complexities’ of those assembled at post‐execution punishment rites too. For, in practical terms the conduct of event management shaped community attitudes to, and expectations of, capital justice, determining whether it was good for government, or not.

Section IV hence explores a selection of representative examples of post‐execution events that worked well from the point of view of medico‐legal officials handling them, and others that did not. We will also be encountering situations in which what was intended turned into another sort of performance event. Some penal operations seemed to threaten the fabric of the social order. Equally, officials could be confounded by the muted reactions of their audiences. This unanticipated outcome generally reflected how the convicted perpetrator had committed a heinous murder (rape, child‐killing, or domestic violence), angering those assembled. On these occasions, they seldom disrupted an extensive dissection, but they still did want to be part of the event staged, and this sometimes caused logistical problems on location. Overall, it was the unpredictability of the public display of the corpse in theatrical spaces opened up, often on a temporary basis, to so many in the community which troubled the forces of law and order when ‘justice was remade from the margins’ by the Murder Act from 1752 to 1832.^13^

## II

The history of dissection has been revitalised in the last twenty‐five years.^14^ It has developed from an inward‐looking study concerned with the professional ranks of medical men and their controversial involvement in illegal grave‐robbing, to a more sophisticated scholarly engagement with the social history of dissection and the cultural mirror this constituted in the changing European Enlightenment.^15^ Revisionists now stress the pivotal transition from ‘old anatomy’ (the study of creation) to that of ‘new anatomy’ (remapping morbid pathologies) – and, in particular, how far dissected criminals came to be understood in more‐than‐anatomical terms in England under the Murder Act. Historical archaeologists, such as Sarah Tarlow, have highlighted, too, how ‘the fleshy realities of death in the modern world are generally hidden’ but that ‘this process began in the eighteenth century’.^16^ There was, according to Tarlow, an inherent socio‐cultural shift that occurred in the mid‐Georgian era. On the one hand, ‘mourners tried, as far as possible, to avoid direct confrontation with anything’ that had close personal associations to them as an individual ‘that might remind them of the actual bodily fact of death’. On the other hand, in the case of criminals convicted of murder, there seems to have been greater interest generated about dissection as a social practice. This was because the act of homicide depersonalized and thus distanced audiences from being implicated personally in anatomical death.^17^ Henceforth the display of the material body started to be reimagined in spatial terms – ‘inner subjectivity was visible externally as part of the *medical gaze*’.^18^ As a result, ideas about the body became ‘multimodal … invoked in different contexts’ with diverse meanings that are central to this article's novel approach.

There are nevertheless very few studies which have been undertaken of the social geography of anatomical punishment venues outside London from 1752 to 1832. And this scholarly neglect is significant because during the period ‘understanding and acting upon the dead body … could involve concepts of social display and performance’ not simply powerfully in transition but contradictory too.^19^ It was feasible to fear a violent murderer at a murder trial and then be powerfully attracted to their storyline at a dissection. Criminal notoriety generally attracted considerable publicity in death, but for the forces of law and order to attempt to control this instrument of deterrence was no easy matter. Consequently, a collage of experiences (moral, religious and material) became embedded into the cultural reception of criminal dissections during the period. Since elements of crime and justice were intrinsic aspects of that social reception, it is important to explore where rituals took place and how they might have shaped notions of power and agency for all those involved. Appreciating then how the spectacle of dissection was more‐than‐anatomy involves first engaging with how exactly each event was socially constructed. Helpfully, social theorists have provided an intellectual framework to rethink in a multidisciplinary manner the nature, purpose and spectrum of post‐mortem ‘harm’: our central focus. Three approaches in particular are instructive about the impact of social discourses attached to capital legislation. For, as Pamela Graves reminds us, post‐mortem penalties became rooted in communities because these were ‘built up through social practice, cultural horizons of meaning, symbolism, and attributes in … social worlds’.^20^

Henri Lefebvre's *La Production de L'Espace* (1974, translated 1991), ‘remains the most comprehensive effort to develop a theory of *social space*’: an approach very relevant for this article's central focus.^21^ It has often been remarked that his cultural concept of space was ‘dense, difficult, fascinating and immediately influential’. Essentially he argued that space in a society ‘is always *socially‐produced*’ in some respect, and that aesthetic practices with a visual purpose (like anatomy) ‘are at every point bound up with socio‐political and philosophical assumptions’. In the case of the Murder Act, this conceptual slant sets in context why post‐mortem ‘harm’ exemplified a cultural consensus that the sinned body should be shamed and that it was reasonable for the state to appropriate northern European religious sensibilities to do so (cutting the dead body being taboo for many Christians). Cultures of stigma were to be exploited in the name of good government; better to cut out the canker at a criminal dissection than see the body politic rot. It was not inevitable that these anatomical gestures would work, but it was very likely they would have cultural purchase given the social construction of the society that passed the new capital legislation in 1752. If we accept then Lefebvre's premise that social space is always ‘produced’, the complex ways in which medico‐legal officials in charge of post‐execution productions undertook venue management of those punishment events evidently merits more detailed scrutiny in crime historiography. Before doing so, however, it is necessary to consider Michael Foucault's thinking too.

Lefebvre, like many contemporary social theorists, was influenced by the intellectual climate of Foucault's 1960s work on the meaning of power and punishment. In particular, his lecture ‘Of Other Spaces’ given in 1968 proved to have remarkable intellectual longevity and multi‐disciplinary application.^22^ Foucault essentially argued that ‘a whole history remains to be written of *space* which would at the same time be the history of *powers*’. As David Wiles explains, these powers would range ‘from the great strategies of the geo‐political to the little tactics of the habitat, institutional architecture from the classroom to the design of hospitals, via political and economic installations’.^23^ His intention had been to start a debate about ‘a range of cultural institutional and discursive spaces that are somehow *different*’ in society. He called these ‘Other Spaces’ heterotopias and urged cultural historians to relocate them. A heterotopia has thus often been thought of as an ‘alternative space’, conceptually somewhere that is different from the norm in society but resonates with the actual world that people inhabit. Heterotopias have consequently been regarded by cultural geographers as places that have facilitated ‘disturbing, intense, incompatible, contradictory and transforming human experiences’.^24^ For the purposes of this article's focus on deterrence, dissection and display, it is the choice of post‐execution premises annexed under the Murder Act that appears to align closely with Foucault's spatial thinking. Their status as ‘counter‐sites’ of punishment performances has the potential to frame new ways of thinking about the social role of penal spaces in provincial society from 1752 to 1832 for England.

Thus (as we shall see below) whilst a law court might function under normal circumstances to condemn a homicide perpetrator in front of a Grand Jury at the local Assize, it could also be requisitioned by medico‐legal officials to double‐up as a venue for dissection. Essentially, the socio‐political space of law and order became a temporary ‘counter‐site’ of post‐mortem ‘harm’. The doors of the premises opened to do a dissection and then closed once it was finished. In the meantime, the acquisition of the social space and the production of the penal rites in it could be staged over several days. This made it a radical forum, a theatre to be culturally absorbed, passing into customary practice and social memory. Indeed, dissection venues appear to have had the remarkable capacity to reflect nearly all of the common features of heterotopias that Foucault outlined:

They would:

1. be established in all cultures but in diverse forms (especially as sites of ‘*crisis*’ or later ‘*deviation*’);
2. mutate and have specific operations at different points in history;
3. juxtapose in a single space several incompatible spatial elements;
4. encapsulate spatio‐temporal discontinuities or intensities;
5. presuppose an ambivalent system of opening/closing, entry/exit, distance/penetration;
6. have a specific operation in relation to other spaces as, for example, *illusion* or *compensation*.^25^

Generally there was a ticket‐entry system to the most popular dissection spaces (recounted in more detail in Section IV). There would be entry/exit points signposted to manage crowd control. Over the course of the Murder Act, different concepts of the body in parts and its morbid pathologies were staged in different ways so that ‘spatial‐temporal discontinuities and intensities’ were acted out side by side for punishment reasons on dissection tables. This was particularly noticeable in the 1790s in the transition from ‘old anatomy’ to ‘new anatomy’. Likewise, because of the variety of venues that were requisitioned the penal rites appear to have been all about ‘juxtaposing in a single space’ what seemed like ‘incompatible spatial elements’, giving rise to the *dangerous dead*.^26^ This term of derision entered common parlance because not all criminals expired on the gallows. Everyone condemned to die had to be double‐checked for life‐signs when cut down from the hangman's rope since they were often capable of being revived. The *dangerous dead* thus threatened the social fabric of the post‐execution penal space itself. Mutability was therefore inherent in deterrence, display and dissection, and had wide appeal for the assembled crowd: again elaborated with archive material later. There was the fearful but enticing prospect that something taboo, foul, stigmatized and deviant was about to take place to which each had public access, and which required some form of compensation (physical retribution), as well as illusion (the dead might threaten to return if the hangman had been inept in the winter cold). It is characteristic of ‘Other Spaces’ that all of these features will be apparent in some respect when we encounter this article's new sources. And this is why it is argued here that what we are about to engage with could be described as *heterotopias of dissection*. There is though a third germane element of social theory too.

David Wiles argues that it is important to keep in mind that there was a tradition in eighteenth‐century playhouses of permitting the audience to pay for an extra ticket to set on the stage with the actors. He explains that today scholars like Mike Pearson are keen to relocate such intimate performances whether ‘inside or outside the institution of theatre’ inspired by Enlightenment drama. One key example of this trend is the growth of immersive theatre in which the audience does not sit in fixed seats, but walks or runs with the actors through a performance space, feeling part of the action. Wiles thus comments: – ‘I want to find different arenas for performance – places of work, play and worship, where the laws, and by‐laws, the decorum of learned contacts with theatre can be suspended. I want to make performances that fold together, place, performance and public.’^27^ Thus just as Tarlow has identified that new concepts of the modern body have their historical roots in the eighteenth‐century world of crime and punishment's changing views of death, so too concepts of avant‐garde theatre could be said to have once been derived from the experiential elements of dissection spaces authorized by the Murder Act. Over time, these penal performance spaces became assimilated into voluntary hospitals, but the key point to appreciate before we encounter representative examples is that they did not start out that way in 1752. From their inception in community life, they closely resembled elements of experimental theatre whose *raison d’être* is to alter conventions of space, theme, movement, mood, tension, language and symbolism. Again Wiles sets this enduring appeal in context:

When I enter an empty theatre, I feel a surge of anticipation, sensing the potential for intense human contact. But … when I watch a play in a theatre, I have the habit of nodding off … The play as text can be performed *in* a space, but the play‐as‐event belongs to that space, and *makes the space perform* as much as it makes the actors perform.^28^

In what ways then were criminal dissections setting the standards for theatrical productions in ‘Other Spaces’ in which ‘the social was performed’ for penal purposes? How exactly did those spaces perform on behalf of the criminal justice processes of the Murder Act? Did event management by medico‐legal officials engage theatrically to make those spaces perform in dramatic ways that impacted on the reception of the capital code and its deterrence value? To begin to answer these fundamental questions in crime studies, we need now to examine the types of venues used on location.

## III

**Figure 1:**
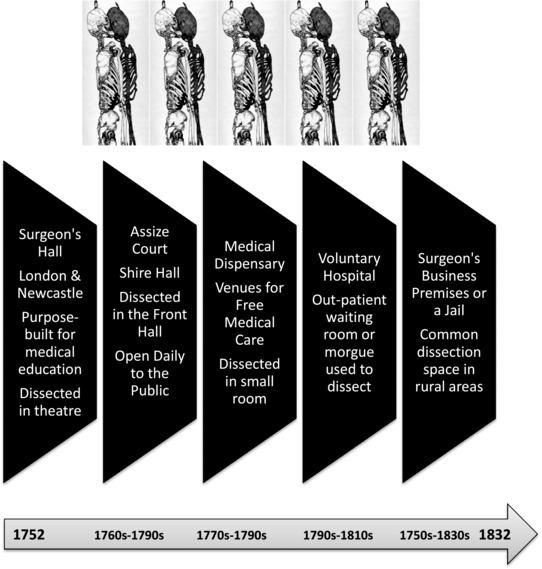
Heterotopias of dissection, *c*.1752–1832. *Source*: ©Author designed, 2017

This article's central focus is those community venues requisitioned by medico‐legal officials outside London under the Murder Act. Our focus is on the criminal dissections ordered by a judge in court and not reprieved before the Assize left town.^29^ To engage with those perpetrators executed and handed over to the medical fraternity, a new dataset of 1,166 homicide cases has been compiled from Sherriff's Cravings raised around England. These were financial records kept by sheriffs in charge of executions and allocating dead bodies to penal surgeons. Any expenses incurred in the course of medico‐legal duties could be reclaimed from central government where they remain archived today. Financial records thus provide details of 923 homicide cases where the judge stated unambiguously that the intention was to send the condemned for a criminal dissection in England from 1752 until 1832 (see Table 1 below). And of those 923 cases, some 230 have been put aside so that this study is not London‐centric, as is so often the case in crime studies of the period.^30^ Instead, it concentrates on a representative sample of 693 provincial cases (64.5 per cent) generated outside the metropolis covering a total of 36 counties. Of these, 102 convictions (16 per cent) where the source material is very rich have been subjected to intensive study. These cases are largely representative of the Midlands area (rural, semi‐rural, industrial and semi‐industrial communities) and have undergone extensive record linkage work.^31^ Before concentrating on symbolic cases, however, it is first essential to geographically group together the sorts of dissection spaces identified in the original record‐keeping for all areas of England, as illustrated in Figure 1.

When the Murder Act was conceived, the legislation was written in rather general terms to allow for some medico‐legal flexibility. In practice it became framed by, first, *social death* (tried and condemned in court for homicide), then *legal death* (hanged in public), followed by *post‐execution ‘harm’* (gibbeted, or a criminal dissection). As I have established in previously published work, there were two ways in which the post‐execution rites were carried out by penal surgeons.^32^ Since the question of when exactly death occurred in the heart–lungs–brain underwent a significant transformation in medico‐scientific thinking during the period of the capital code, penal surgeons had to adjust to a mutable material reality when conducting post‐mortem ‘harm’.^33^ They often observed that not every prisoner died on the gallows. Some hangmen, as Owen Davies and Francesco Matteoni have investigated recently, were inept.^34^ If the rope slipped around the windpipe then the murderer might cheat death. Other medico‐legal officials discovered that, in the winter cold, bodies that looked dead could be revived: today, this is known as the science of cold therapy and it is often deployed in trauma cases when the blood is highly oxygenated but the body is placed at very low temperatures to keep the injured alive in resuscitation medicine. In the past, the discovery that this was medically feasible (an accidental outcome but one that changed medico‐scientific opinions) meant that penal surgeons adopted a new choreography to handle any physical anomalies that occurred whilst the condemned body was in their jurisdiction. Essentially, they reversed the post‐execution wording of the Murder Act. Instead of being hanged and then ‘sent to be *dissected* and *anatomized*’, surgeons first *anatomized* the body – which meant cutting it open with a crucial incision, cross‐like from the neck to the navel and across the chest – to check that medical death had taken place in the heart–lungs–brain – and only then did they proceed to a full‐scale *dissection*. In order, however, to choreograph these two post‐execution stages of punishment with proficiency, the choice of venue where these procedures were designated to take place was a constant anxiety.

The location needed to be near the gallows, otherwise the body would rot before it could be dissected. It had to be able to accommodate the penal surgeon, any apprentices, and a dissection table. Only a spacious auditorium could hold back the crowds and give the penal surgeon scope to check on the living status of the condemned. Many local people walked beside the criminal corpse (usually conveyed in a cart) to witness its dissection. And so, the surgeon needed elbow room to be able to do an initial examination before the crowd pushed forward for a better view. To get people in and out of the venue efficiently without causing a riot there thus had to be an entrance and exit of some sort, otherwise pushing and pulling during the ingress and egress of everyone assembled could have caused a stampede of spectators. For the display and spectacle to make a cultural impact in the community it always worked best if a local Shire Hall law court doubled up as a criminal dissection space. And this is why it was one of the most common types of premises used in the sample size of 693 cases (64.5 per cent). Thus, whilst London and Newcastle from the 1760s each had a bespoke anatomical theatre specially built for criminal dissections, these were not typical of the venue choices for post‐execution punishments in the provinces (as again Figure 1 shows). An emblematic case study from Chester is illustrative.

On 21 August 1812 two prisoners were convicted of ‘wilful murder’ in the Shire Hall at Chester Castle.^35^ John Lomas, a farmhand (aged 17) and Edith Morrey, his married mistress (aged 32), had together killed George Morrey. Edith's husband had been bludgeoned to death with an axe. Under cross‐examination Lomas confessed to premeditated murder. The homicide, he alleged, was perpetrated to enable Edith to claim her widow's inheritance. The lovers planned to marry and reside at the family farmhouse. In court Lomas showed remorse, but Edith maintained her innocence. Three months prior to the murder she had miscarried. This, it was argued, deranged her mind. Edith was mentally incapable of coordinating a murder plan; a jealous immature lover had acted alone. After closing arguments, the jury rejected that defence. Edith had wilfully committed adultery, inveigled her lover to murder, and tried to evade justice by stabbing her throat with a razor on the night of her arrest. The accused were sentenced to be hanged on the gallows and then to be handed to a surgeon for dissection. This tale of the alluring murderess, her foolish lover and six surviving fatherless children evoked considerable moral comment in the provincial press. Newspaper reporters covered the death and dissection penalties in full. Publicity surrounding the case thus reflected sustained local interest by ordinary people in attending the entire spectacle of punishment – in court, at the public hanging and processing with the corpses to the chosen post‐mortem premises.^36^ This timetable of punishment, moreover, was not exceptional, despite the notoriety of the protagonists. Their punishment journey had to be managed and timed judiciously from the scaffold to the dissection venues by the medico‐legal officials. The case is therefore instructive of how important venue management choice was when it came to the display of the criminal corpse (in this case, two prisoners on two separate occasions). Events highlight the fluid nature of the multi‐purpose premises that were used, both here and repeated often elsewhere.

The judge ordered John Lomas to be executed within two days of sentencing in court and then dissected over another two days, a total of four days of physical punishment: the average timeframe. Even so, seasonal timings in all capital cases caused penal surgeons to wrangle with decomposition rates: a common time constraint. In winter the corpse rotted slowly, whereas in summer putrefaction took just four hours. The spectacle thus occurred in the centre of the community and remained a public event until the body shell was despoiled. Meanwhile, Edith, the co‐accused, anticipated being sentenced for ‘petty treason’ and to be burned (the penalty for a spouse‐killer), but the judge ordered her to die on the gallows and be dissected too for being conniving. Edith's defence council then made a dramatic intervention that she was: ‘quick with child … these five months’.^37^ The judge ordered Matthew Hudson, Chester County Gaol governor, to have Edith examined. Owen Titley, a Cheshire surgeon, was to monitor her pregnancy officially, and report back to the court.

Four months after John Lomas's execution Edith Morrey gave birth in Chester City Gaol to a healthy boy on 21 December 1812. Local newspapers reported that she hoped for a pardon so her seven children would not be orphaned. Samuel Burrows the Cheshire hangman was not minded to be merciful to a murderess. As instructed, he executed Edith at the spring Assizes of 1813 and sent her baby for adoption. *The Times* newspaper described how ‘ten thousand people’ assembled for her execution and post‐mortem punishments, timetabled in tandem:

On Friday morning at 12 o'clock this wretched woman was delivered by Mr Hudson, constable of Chester Castle into the hands of Messrs Thomas and Bennett, the City Sheriff for execution …; she got into the cart, and immediately laid down on one side, concealing her face with her handkerchief, which she had invariably done in public … She was very much convulsed for a few minutes when her pangs ceased in this world. After hanging the usual time, her body was delivered to the surgeons for dissection, and was open to public inspection during all Saturday.^38^

It is evident that the Murder Act had captivated the public attention of Chester people who congregated at the hanging‐tree. Yet, the post‐execution punishment rituals attracted an avid audience too. In this case, Edith Morrey's body was hanged at Chester Gaol and then sent next door to Chester Infirmary. Owen Titley initially opened ‘her [Edith's] thorax and abdomen and removed her heart for preservation’ (he had done the same for Lomas).^39^ The penal surgeon was effectively checking that the prisoner was dead and had not survived the gallows.^40^ Then he sewed the body back up and sent it back to the Shire Hall courthouse. On the way it was accompanied by a huge parade of local people. Edith's body was brought inside the main entrance and placed on a portable dissection table. The public space was carefully chosen in the community both for its legal symbolism and for the practical reason that it could accommodate the large audience assembled to see the post‐mortem cuts. In the main hall, the body was left open for public consumption over the next twenty‐four hours. Local people filed past the criminal corpse in two orderly queues. The throughput of audience members was calculated by local newspaper reporters to be ‘four hundred and sixteen people per hour’ – on average seven spectators a minute – to ensure that ‘all ten thousand spectators’ had a chance to view the criminal corpse. They were permitted to look but not touch, and were directed to keep moving along Edith's cadaver, down two sides of the dissection table. The next day, the Shire Hall doors were closed, and the criminal corpse was moved back to Chester Infirmary to complete a full‐scale dissection conducted by Owen Titley. In this emblematic case, we can begin therefore to see how dissection was more‐than‐anatomy – it was about the choice and organization of venue management in social spaces that exuded judicial authority, medico‐legal power and the crowd's agency, capable of containing changing notions of death and its liminal boundaries. The evidence also points to a series of theatrical acts on multiple stages indicating that post‐execution premises have been over‐simplified in standard historical accounts.^41^ The Chester example hence includes two types of venues often requisitioned, again as in Figure 1. And whilst these changed over time, they could also overlap for reasons of convenience. There were likewise potentially three phases to each post‐mortem spectacle in the provinces and each chosen venue had to have adequate facilities to pace the tempo of the dramatic denouement. It is this changing punishment scenery and its event management by medico‐legal officials which still merits closer historical scrutiny.

Provincial penal surgeons often exploited their discretionary powers under the new legislation to manipulate access to, and the time‐consumption of, the criminal corpse in their chosen venues. Hence in a similar manner when William Voce was found guilty of the murder of an ‘unfortunate widow named Mary Dufty’ on 16 March 1775, the judge at the Nottingham Assize sentenced him to be executed according to the Murder Act within two days. This was done outside the courtroom where he had been convicted. The gallows was erected in the forecourt of the Shire Hall of Nottingham city‐centre. Then at noon, as the parish church‐clock opposite struck, a street procession began. It came full circle back to the courthouse within the hour. Effectively, the condemned was being processed to call residents to engage with his spectacular end. Once executed, the condemned body was taken down and brought inside the courtroom premises for a public dissection. The court‐house did not have a purpose‐built anatomical theatre. Instead Nottingham surgeons requisitioned the largest room off the front hall of the law courts. When the body arrived it was therefore still warm, and, as we shall see in Section IV, this created the opportunities for certain types of experiential encounters with the criminal corpse, influencing its potential deterrence value. The costs meanwhile accrued are instructive about the punishment timing:

Paid for man and horse to go for the hangman to execute Voce £0, 5s, 0d

Paid hangman £1, 1s, 0d

Paid for Cart Horse &c, £0, 10s, 0d

Received …

Five Pounds Eleven Shillings (£5, 11s, 0d) Charged for Attending the Dissecting of Voce.^42^

Thus it was reported by the Sherriff in the case of William Voce:

At ten, he was brought up from the condemned cell, and had his irons knocked off, and at twelve the procession set out, and arrived at the fatal spot within the hour. After the usual hanging time, the body was conveyed to the Exchange‐room [of the courthouse], and being extended on a table, was inspected by great numbers. It was then subjected to dissection in the same place.^43^

In similar circumstances when ‘James Brodie aged 23 was arraigned’ at the Shire Hall in Nottingham on 12 July 1799 for the ‘wilful murder’ of ‘a boy named Robert Henesal’, there was ‘great excitement’ to see his post‐execution spectacle. It was reported in local newspapers that: ‘His body was … afterwards publicly exposed … Crowds of men, women and children, indulged their morbid curiosity, by thronging the scene of this most repulsive spectacle.’^44^ Aside from the sensationalist tone, the observation by newspapers reporters present that ‘some 10,000 persons were in attendance’ is instructive. Even by 1815 when dissections had moved to a new local voluntary hospital built in the vicinity of the Nottingham gallows, the size of the crowd had not dissolved. So much so that when ‘John Hemstock alias Black’ was convicted at Nottingham Assizes for the ‘wilful murder’ of James Snell, a servant to Mr Wells, farmer of Clarborough near Retford, his dissection attracted ‘immense’ spectators of ‘not less than 5,000 in attendance’. What seems to have motivated a great deal of anger in this third case was the fact that the homicide method had been brutal. Hemstock ‘murdered the lad by striking him on the head with a bar of wood, and then cutting his throat with a razor’. Although after the death sentence was passed ‘he begged most earnestly of the Judge to remit that part of the sentence which ordered his body to be dissected’, no clemency was shown. His criminal corpse was reported to have been showcased twice, first at the Shire Hall and thence ‘taken to the General Hospital for dissection, and exposure, where the skeleton, hung on wires, is still in a good state of preservation’.^45^ In all three representative cases there were self‐evidently ‘very sizeable crowds’ to manage and so the nature of the event management in Nottingham city‐centre was choreographed in a manageable convenient venue (even allowing for some reporting embellishment to increase newspaper sales). Indeed of the thirteen convicted murderers sentenced to dissection in the official Assize records for Nottingham, ten underwent the same public post‐execution punishment timetable involving the Shire Hall court house in the 1790–1820 period (only one would be reprieved; two were hung in chains, as in Table 2). These numbers might seem small compared to the number of the poorest dissected under the Anatomy Act after 1832, but their symbolic importance in the community should not be underestimated either.

**Table 2:** Convicted Homicide Cases, executed and dissected: Midlands Court Assize Circuit, England, 1752–1832

Midlands Court Circuit	Number sentenced to dissection	Number pardoned by the judge	Number hung in chains	Number of suicides	Total Number of murder convictions	% of all Murder Case(s) Dissected
Derbyshire	9	1	2	0	12	75%
Leicestershire	16	1	2	0	19	84.2%
Lincolnshire	20	2	5	0	27	74.1%
Nottinghamshire	10	1	2	0	13	76.9%
Northamptonshire	13	1	0	0	14	92.9%
Rutland	1	0	2	0	3	33.33%
Warwickshire	33	2	7	0	42	78.6%
**Totals**	102 cases	8	20	0	130 cases	78. 46% average

*Sources*: The National Archives, Sheriff's Cravings for England, 1752–1832, cross‐checked to Assizes Records, County Record Office(s).

If then the Shire Hall was a popular venue for post‐execution punishment throughout the Murder Act, smaller types of venues were requisitioned too for reasons of convenience and event‐management. In counties like Lancashire and Yorkshire, penal surgeons tended to appropriate medical dispensaries to conduct criminal dissections conveyed from the nearby gallows. The *Edinburgh Gazetteer* of 1828 thus described the buildings in Wakefield (for instance): ‘In Wood Street is the court‐house, the new banks, the corn and auction mart, and that elegant building containing assembly rooms, news‐room, library, a music saloon, and a suite of apartments allocated to officers of the dispensary.’^46^ It was here that executed bodies were distributed if there had been multiple executions on the same day at York because the pace at which corpses rotted dictated what the hangman decided to do if the penal surgeon on duty could only practically dissect one body at a time before putrefaction. After double and triple hangings therefore it was better to distribute surplus criminal corpses close‐by to a township like Wakefield or its companion town Halifax. Michael Pickles was one such criminal corpse. He was arrested for murder after a burglary went wrong at Hebden Bridge in 1817. Arraigned for the murder of Samuel Sutcliffe, Pickles and his accomplice John Greenwood were arrested and sent for trial at York Assize on 14 March 1817. The *Leeds Mercury* reported on how they had robbed the home of Samuel Sutcliffe but regrettably the house burglary had escalated into homicide when a shot from a gun was fired with fatal consequences.^47^ It was unclear who had pulled the trigger and so the jury passed a guilty verdict for each of the accused. Accordingly both were hanged at York, but Pickles's corpse was sent to Halifax dispensary for dissection to the West Riding of Yorkshire. This was a symbolic act – near to where the original murder had been committed – and a practical gesture – in York the body would go rotten too quickly to be event managed. The dispensary for the sick poor had popular appeal in the community because it provided free medical care via subscribers. To conduct a criminal dissection in this location thus encouraged the local populace to endorse a post‐execution event that they might not otherwise have been able to travel to in York. Its event management would however in logistical terms have been a very different form of theatre than that at a large Shire Hall. The room set aside in the dispensary premises was much smaller, and therefore it was important to ticket‐time everyone's entry and exit. To engage comparatively with this set of spatial considerations, examining contemporaneous circumstances at Winchester is instructive.

On 28 July 1812, a violent murder was reported across the country, and carried by Irish newspapers.^48^ A young man named ‘John James aged 19’, an apprentice to a shoemaker, was charged with the murder of ‘his mistress, Elizabeth Hill’. On Sunday 21 June 1812, the young apprentice's master had gone to church, and whilst he was out of the house the lad took the opportunity to kill. On his return Mr Hill found his wife dead, ‘lying on the kitchen floor, with three deep wounds inflicted with a hatchet to her head and face, and her throat cut across’. As the homicide had taken place at Shalfeet on the Isle of Wight, it was decided to try the case at Winchester court in Hampshire to ensure a fair trial. To the shock of the assembled jury, the accused showed little remorse. Newspaper reporting described his demeanour in court as ‘he calmly confessed the foul deed … with his eyes bent to the ground in a melancholy apathy … he heard the awful sentence’. Indeed, the judge, Sir Alan Cambre, commented that the convicted John James was not only dangerous but had a tendency to believe that the Bible authorized the dreadful murder, for he kept repeating passages from the ‘third chapter of Job’ in his defence. The theme of his remarks was that his birth was cursed and he deserved a life of darkness. Cambre, in passing the death sentence and dissection afterwards, warned those assembled about ‘the dangerous effects of vulgar and literal conceptions of scriptural passages’. It was thus reported that on

Monday se'nnight [sic] about eight o'clock in the morning, John James, convicted at the late Winchester Assizes, of the wilful murder of Elizabeth Hill, at Shalfleet, in the Isle of Wight, was taken from the county gaol, to the usual place of execution, and after spending considerable time in devotion with the Ordinary of the prison, was launched into eternity – After hanging the usual time, his body was taken to the County Hospital for dissection.^49^

The Hampshire County Hospital was founded in Colebrook Street Winchester in 1737 and according to its building records had a commodious ‘dead room (*a convenient place for receiving a dead body*)’.^50^ Soon, however, because of the rapid expansion of the city's population, bigger hospital premises were needed; the number of tourists passing by from Southampton docks, and rising numbers of sick pilgrims visiting the relic of St Swinthin's bones at Winchester Cathedral had stretched local medical services too. Clobury House on Parchment Street just off the High Street was therefore refurbished as a more commodious hospital in 1759.^51^ It was here that the medical staff dissected criminal corpses under the Murder Act. Their ‘dead room’ had the capacity to accommodate a cold storage morgue with a waiting area for the general public and a small medical museum located next door to display the dissected.

The condemned man named John James was thus brought from the city‐centre prison located on Jewry Street in 1812. He was taken up the hill on an escorted five‐minute walk to the Assize Court at the Great Hall. There he was tried, found guilty and executed outside the main door by the Old Castle walls, before being carted down the hill to the County Hospital just a short sojourn away on Parchment Street in the city‐centre. This meant that the topography of the punishment journey worked with gravity: for practical reasons it was judged better to walk the condemned up the hill and, once executed, cart the heavy dead corpse down again on its post‐execution rite (commonplace in many hanging towns). At the same time, the execution crowd numbering ‘above 5,000’ that accompanied ‘John James the accused’ to the Assize court room could be dispersed conveniently after the execution because the hospital site on Parchment Street was just a stroll from a large public space in front of Winchester Cathedral. If those attending the post‐execution event exceeded the capacity of the County Hospital dead‐room where the corpse was due to be dissected, then the queues could spill out down the lane onto the Cathedral Green. This social geography also prevented people from getting bored when standing around or rioting if they felt excluded: themes we will return to in Section IV. They had the option to peel off to visit the inside of the Cathedral until the throughput of people assembled was either speeded up by the medico‐legal officials on duty, or a sufficient number of spectators had been processed through the dissection space to accommodate those waiting longer to get in and see what was happening. This was self‐evidently a very different experience from the one that would be organized typically at a small dispensary in the North of England.

*In situ*, when utilizing hospital premises for criminal dissections there was hence a basic calculation to make in terms of event management everywhere once Shire Halls handed over their punishment function to medical charitable institutions. It would literally take at Winchester (and elsewhere) the ‘5,000 people’ wanting to be part of the dissection display a considerable time to file past. In fact 1,000 spectators per hour could only be stage‐managed by creating two queues lines of 500 people each, and this facilitated a throughput of about 16 people per minute (8 in each queue) down two sides of the dissection table. They each got a hurried view, but the body did not start rotting badly and so could be dissected at the start of day two post‐execution. Nevertheless, as we shall see in more detail in Section IV, some in the crowd wanted more than a glimpse and they often joined the queue again to get a better look a second or third time – or in notorious cases, as much as five times. People at a dispensary session (in the North) or a Shire Hall event (in the Midlands) could often make repeated visits much more easily than at voluntary hospitals (built across the North, South, East and West by 1832). The dissection event was therefore as much about the chosen space performing its theatrical role on behalf of crime and justice as it was about the official conduct of the medical staff on duty. Indeed, the practicalities attached to event management *per se* in hospital premises were akin to immersive theatre today (framed by David Wiles, Section II). Letting audiences appear to be part of the action by letting them be in such close proximity to the criminal corpse displayed for their spectatorship was a powerful incentive to be with the main character in the dissection room drama and by implication its deterrence message: reiterating Foucault's model of the subtle social forms of power at play in these ‘Other Spaces’ of punishment.

If these were thus the main types of premises used and they appeared in one form or another across most of provincial England under the capital legislation, then there was one further aspect of this social geography that needs to be set in context before examining events in more detail below. For, the Murder Act had a lot of timing implications that have seldom been explored.^52^ It is important therefore to keep in mind that ‘time itself, was one of *the* contested terrains of the eighteenth century’.^53^ Paul Glennie and Nigel Thrift thus tell us that there was ‘no such thing as *clock* time’ during the period of the capital legislation:

Rather clock time comprises a number of *concepts, devices* and *practices* which have meant different things at different times and places, and even in one place have not had a single unitary meaning … It is emphatically *not* the case that ‘clock time’ was created by accurate pendulum‐based timekeeping from the seventeenth century (or by industrial work based discipline by the eighteenth) and then has changed little since. Many different clock times have been formed over time and space, by many groups of people; groups which can be conceived of as social networks and communities.^54^

It follows that conducting a dissection in communities in which time was so mutable (shaped by the sun, tides, seasons, religious festivals, church clocks wound to different bells & changing mechanisms) could leave surgeons open to criticism if they hurried or mistimed public expectations at a punishment spectacle. All however depended on the rate the body became despoiled.

What often made criminal dissections tantalizing was their theatrical potential to stage ‘multiple, sometimes, conflicting time‐reckoning systems’ operating in provincial society.^55^ There were four basic mentalities in the 692 cases investigated: *body time* (the natural cycle of birth, life and death); *medical time* (monitoring expiration in the heart–lungs–brain); *rotting time* (rates of rigor mortis, decomposition and putrefaction); and *sacred time* (marking when vital forces left the body shell for an afterlife). This complex context explains why contemporaries believed it was necessary to ‘*bide one's time*’ until the timetable of punishment was reconciled by the penal surgeon on duty, as we saw in our emblematic case at Chester. Yet, these concepts of law and order were further complicated by temporal mentalities that were *timeless* too. These included folk memory associated with miracle cures on the gallows, as Davies and Matteoni have highlighted in documenting superstitions about the healing properties of blood and body‐parts.^56^ In an oral culture retelling notorious criminal tales also gave rise to popular dream stories featuring ghost time, unquiet spirits and eternity, exemplified in Shane McCorristone's studies.^57^ Medical men meanwhile were intimately involved in a temporal process that served the production of new anatomical knowledge for posterity. These Janus features of punishment (looking back to a time when the body expressed a divine creation and yet forward to embrace future anatomical innovation) were all beyond the strict boundaries of conventional historical time and therefore troublesome for the forces of law and order to control. When therefore a presiding judge condemned a convicted perpetrator to die and refused a pardon, the medico‐legal officials were tasked with event‐managing complex notions of time‐keeping that were multi‐linear and multi‐directional as well. Church clocks marking execution timings did not necessarily operate in a linear fashion or regularly. The corpse itself had a non‐linear timing because human beings have bodies that die biologically at different rates. It is important therefore to appreciate that the body‐clock of the condemned person was perceived as a time‐keeping device in its own right, with a ‘heartbeat, life‐course changes, and body morphology’ that still ‘remains under‐theorized and under‐researched in the historical record’.^58^ How then were these aspects of post‐execution punishment worked out on location and with what results? To engage with these important questions that framed the deterrence value of crime and justice it is necessary to examine a selection of cases in greater archive detail.

## IV

The most basic decision that surgeons had to make when they received a body from the gallows, in all 692 cases, was where exactly in the designated dissection space to place it.^59^ This might seem an arbitrary detail but in fact it shaped the visual impact of the display and therefore the wider cultural reception of the deterrence value of the capital legislation. There was little point in punishing the body for public consumption if few local people could actually see it. Whether they came into the designated space for reasons of public curiosity, to witness a major community event about which there would be a lot of gossip, or to ensure that punishment in England remained a public affair (as opposed to European traditions where it tended to happen behind closed doors), the fact remained that many people did want to participate in the temporal and spatial dynamics of the post‐execution event.^60^ It was also the case that as new medical facilities in the community were erected, and travel between counties became quicker and easier over time, more interest was being generated in a flourishing medical press, attracting larger crowds to criminal dissection venues.^61^ Often in newspaper reports the logistics of this common situation were reported. Thus, by way of example, when two Scottish brothers Alexander Keands (aged 35) and Michael Keands (aged 24) attacked a publican's wife Mrs Blears and murdered her servant Betty Bates at Winton near Worsley in Lancashire in 1826 there was a lot of local interest in the capital case. Upwards of 10,000 people attended the execution scene.^62^ At the appointed time, a local reporter noted that Alexander did an odd thing. He ‘took Michael's right hand in his left’. As the rope tightened, Alexander ‘stopped jerking’ within minutes whereas Michael ‘struggled violently’: emphasizing (some thought) the latter's culpability. There was thus a lot of excitement in the town about whether the post‐mortem punishment rites would reveal the true nature of their sinister characters.

Local accounts stressed that ‘several applications from individual surgeons were made to the sheriff for the bodies, but he declined granting any of them’.^63^ This was because he had already allocated one body – that of Michael – to Manchester infirmary and, the other – of Alexander – was given to Lancaster surgeons. In the case of Alexander, a new hospital infirmary had now been built in Lancaster during 1823 and this replaced the medical dispensary at the bottom of Castle Hill in the town where previously the criminal corpse would have been taken for dissection. The change of premises reflected just how much local crowds had grown in the intervening years at criminal dissections. Newspaper reporters noted that the new medical venue was much bigger to accommodate local people but on this occasion nevertheless a serious public order situation had arisen. Medical men tried to do the criminal dissection in private to raise their professional standing. However, in attempting to do so, they badly misjudged the timetabling expectations of the post‐execution crowd:

The body of Alexander was delivered to the infirmary on Wednesday evening and on Thursday morning, the surgeons commenced dissecting it. So great was the public curiosity to see the body, that a great crowd of persons forced their way into the dissection room, and filled it to such a degree as to put a complete stop to the operation [of the law]. The body was then taken into the yard, and exposed, and for some time, to the view of all who chose to go and look at it. The dissection was resumed and, we believe, completed yesterday.^64^

The surgeons neglected to timetable the conventional placement of the criminal corpse, displayed in the middle of a dissection venue to public view. In doing so, the riot that followed revealed the local importance of clear sight lines to the condemned in a communal setting. As a reporter for the journal *The Age* noted in the Sunday 26 August 1826 special edition: ‘it would appear that the townships’ around Lancaster ‘had sent forth their entire population’ to the execution and it was these ‘groupes [sic] of people hurrying’ to accompany the body that presented a public order challenge for the dissectors.^65^ It was though remarked on in local newspapers that those present whose emotions had been aroused did not seek to touch or interfere with the criminal corpse. The populace wanted access to the body to make sure it had paid its legal dues, but remained disinterested in actually handling dissection *per se*: an important distinction.

A related issue to consider was the number of entrances and exits (gates/doors) to the allocated space: as we saw at Winchester. So for instance if the dissection table was placed in the middle of the temporary space then a basic decision had to be made about how many people could circulate around it in an hour. If there were two doors, one reliable system was to get people to enter by one and leave by the other. This gave them time to circulate but it also was time‐consuming to permit this. It took more time to walk around a body than to parade past it down one side only. If there were four doors in the room – potentially two exits and two entrances – then a parallel system of queuing always worked best. Two lines of people could enter and leave at the same time, doubling the viewing capacity. These logistics for instance worried officials at one of the most infamous criminal dissections of the era, when William Burke was cut open in Edinburgh. The *Caledonian Mercury* on 31 January 1829 reported that their calculation was that the number of visitors to the dissection room totalled ‘some 24,000 … 68 per minute and 4,080 per hour’.^66^ The through‐put of people was managed by permitting the audience to walk past just one side of the criminal corpse and then leave by a stairwell. Those that tried to do repeat viewings had to queue up again: some were deterred by the long wait, others jostled along.

We encounter a similar situation at the criminal dissection of William Corder who committed the renowned ‘Red Barn murder’ in Suffolk twelve months before the Burke and Hare scandal. Corder's body was cut down from the gallows and taken to the Shire Hall in Bury St Edmunds to check he was dead. A lot of spatial detail and its penal timetable were reported on in the local press. This was done to reassure the general public that such a notorious murderer had received the full legal penalty, as the *Morning Post* of 14 August 1828 elaborated:

About half an hour after the execution the body was removed to a private room in the Shire Hall where Mr Creed, the county surgeon, assisted by Mr Smith and Mr Dalton, made a longitudinal incision along the chest, as far as the abdominal parts, and deprived it of its skin, so as to exhibit the muscles of the chest … They were going to move him to the hospital but this was objected to by Mr Foxton (the completer of the law) [hangman] until he had first stripped him of his trousers and stockings [the executioner by custom was entitled to sell these].^67^

The newspaper reporter explained that the first penal duty was to try to accommodate ‘5,000’ people determined to accompany the body on its post‐execution journey. Their actions reflected a strong public reaction to the infamous murder. The reporter continued:

The anxiety to see Corder's body was as great as at his execution: crowds flocked around the doors. He was put in the Nisi Prius Court, and the people entered at one door and departed at another; he was placed on the table, and, with the exception of his trousers and stockings, he was naked; there was not much change of countenance, but it appeared there was a great affusion [sic] of blood about his throat. Such was the anxiety to see him that we heard several females boasting that they had been in to see him five times after his head was shaved!^68^

Such excerpts confirm general findings already presented in this article and underscored by some 692 cases. There was a lot of public curiosity motivating people to attend the post‐execution performance of retributive justice. The community was evidently determined to accompany the corpse from the gallows. In terms of the event management of the penal choreography, the execution and its criminal dissection were crowd pleasing events for those assembled at Shire Halls in provincial life. They moreover resembled the format and timing of classical Greek tragedy that Aristotle espoused:

Tragedy, is then, an enactment of a deed, that is important and complete and of magnitude, by means of language enriched [with rituals], each used separately in different parts [of the play]; it is enacted, not [merely] recited, and through pity and fear it effects relief (catharsis) to such emotions.^69^

Evidently whatever system of moving people through the penal space was chosen, it was essential to give each spectator enough time to view the corpse to engage their emotions in some respect. Those that were rushed walked outside and came back again, sometimes as many as five times in a single day. Repeat viewings often created timetabling problems. Another key example is again typical of the 102 cases investigated in the Midlands.

On 29 March 1788 the *Leicester and Nottingham Journal* reported on the execution of Thomas Grundy sentenced to death and dissection for poisoning his brother John Grundy at ‘Dale Abbey in Derbyshire’.^70^ At the hanging‐tree the crowd of spectators from the surrounding area was described as ‘very numerous’ exceeding ten thousand. As the murderer was ‘in his 20^th^ year’ and had a strong muscular body, there was a lot of local anticipation about the dissection to follow. In due course his condemned body was ‘brought into the Derby County Hall [Shire Hall] where it was ‘publicly dissected in the presence of a great number of spectators’. The penal surgeon therefore accommodated the criminal corpse inside the courtroom main entrance and ensured that the public doors were left open for forty‐eight hours so that ten thousand people could ‘look, and look again’ however brief the glimpse. These timescales are informative because although the cold spring weather would help preserve the body, after a forty‐eight hour delay there was a greater risk of putrefaction. The fact then of adopting this timetable appears to confirm how much local people could determine the timing of local law and order, even if it risked rendering the corpse less useful for a subsequent full‐scale criminal dissection.

One of the difficulties, of course, with assembling notorious criminal cases is the question of representativeness. It is essential to try to test what happened at criminal dissections in areas of the country that tended to organise post‐execution punishments in low key venues too. Typically this happened across rural areas like East Anglia or the West Country for logistical reasons. Roads across Cornwall, Dorset, Norfolk and Somerset were often very muddy in winter. It might take two days to convey a body to a major medical centre like Norwich or Bath for a criminal dissection. And often it would have been a wasted effort as the body in question would already have advanced putrefaction by the time it reached its destination. These communities tended not to have, and so preferred not to travel to, a substitute Shire Hall. It was commonplace therefore for these criminal corpses to be dissected in the gaol room or the business premises of a trusted local surgeon. This did not however lessen ordinary people's interest in the post‐execution rituals: quite the reverse. In an article of this length only selective cases can be cited but they are nevertheless instructive of rural trends in less accessible places.

At the dissection of Elizabeth Morton on 7 April 1763 for murder at Calverton village a parish surgeon prepared her corpse for public inspection. In early spring the local roads were mud‐ridden. Nottingham Shire Hall, seven miles north east, was judged to be too far away. The cadaver was thus opened on location to check it had died on the gallows and then was exposed for forty‐eight hours at the penal surgeon's house. A local reporter was in a position to write that: ‘her features were rather attractive than repulsive: she was strongly made, and tall, considering her age … 18’.^71^ Age and gender were factors in the degree of public curiosity stirred up locally. So too was the agency that ordinary people expressed in their physical presence, exuding a rural sense of community and belonging. In a similar refrain when Betty Marsh killed John Neville of Morden by bludgeoning him over the head with a blunt instrument in East Dorset, her execution was staged at Dorchester County Jail on 17 March 1794.^72^ The corpse was handed to two local surgeons to dissect. John and Philip Coombes opened the body for public inspection at their business premises because Bath (the nearest medical centre) was too far afield in an inclement season. The doors were left ajar for twenty‐four hours and they then took the opportunity to complete the dissection of the fourteen year old female, enhancing their medical credentials in the community. Further away in Cornwall prisoners, like William Burns, convicted of murder in December 1814, expected to be incarcerated in a place where they would be dissected.^73^ At Bodmin Gaol, Joseph Hamley a local surgeon from East Cornwall often staged the post‐execution rituals in a room at the prison left open to the public for a minimum of twenty‐four hours.^74^ In all these cases, the number of local people that had to be accommodated was a lot less than in a Midlands location like Nottingham Shire Hall. Nonetheless the logistics were essentially the same: the body needed to be centre‐stage, there had to be enough space to accommodate a people‐flow in an orderly manner, and this public access was designed to satisfy considerable levels of sustained community engagement.

Reflecting then more broadly on the 692 cases compiled for this article, the balance of the evidence indicates that increasingly medico‐legal officials were uncertain about how to manipulate the logistics of time spent at criminal dissections to enhance their visual appeal. When in places like Leicester for instance it was very common to have crowd control issues of upwards of ‘fifteen thousand people’ trying to enter a dissection venue there were serious event management concerns being expressed. By 1816 the governors of the Royal Infirmary who provided a room to dissect thus stated openly in their minutes that ‘it would be *absolutely necessary* to have a strong guard’ from now on.^75^ From 1752 they hired a constable to protect the premises of local courts. Yet, around 1815 so large were the audiences assembled at the city's voluntary hospital – where criminal dissections had been moved – that ‘four constables’ were paid to manage the logistics. In the event of a stampede, this was for the ‘protection of the garden and walls … for the prevention of mischief and depredation’.^76^ Together the officials on duty henceforth tended to take a set of logistical decisions that were about creating punishment opportunities for not passive, but active forms, of penal spectatorship. The idea being to give the sense impression of better access, without lengthening the actual punishment access any longer than was necessary.

To do this, the staging had to create an illusion that ordinary people were even closer to the criminal corpse than ever before. Once hospital venues had to accommodate enlarged audiences, a revised floor plan was introduced. The design was all about trying to make the visual experience more vivid. One key architectural change was the replacement of a portable dissection table with a permanent one, carved from marble or redesigned in wood. Hence at Devon, Leicester and Manchester Royal Infirmaries a master craftsman was commissioned to make a new table that could also rotate to make sure everyone could see the many faces of death.^77^ Above it were hung chains, jacks and hoists, vividly lit, encouraging the audience to look up once the corpse was on a pulley above their heads. So inside a hospital, the facilities and equipment often made it feel more intimate than a large Shire Hall space. The aim was to encourage people to talk animatedly about the corpse being opened up, and express emotions about the materiality of the experience. Since those present still could not feasibly spend longer than a minute or so with the corpse, the performance of criminal justice had to be about creating a link between the legitimacy of cutting up the condemned and the spectacular vividness of what the audience saw: the latter gave the crowd the just cause and punishment reason to physically let go of the criminal corpse as they left the room.^78^ Once outside the dissection space, gossip about what had been seen and its symbolic meaning could then be pooled to become in time a community memory. In an oral culture it has often been very difficult for historians to document these absent conversations, seldom written down for posterity. High‐profile cases do however provide important insights – what Wiles has called ‘creating meaning from the scattered debris’ of ‘*what once was*’.^79^

This article has already referred to the infamous Red Barn murder by William Corder in Suffolk. It is well‐documented that it stirred up the emotions of the large number of spectators fascinated by the case and its criminal dissection. Yet, record linkage work has not tended to explore what impact the post‐execution event had on audience members that day. And this is a missed opportunity in crime historiography because it did leave a strong impression on one spectator who attended Corder's execution and subsequent criminal dissection at Suffolk Hospital close to Bury‐St‐Edmunds. The facts are that William Partridge killed a young boy called George Ansell aged 10 years old. He did so because the lad was the servant of a farmer at Milden in Suffolk and he accused Partridge of ‘having stolen a lamb cosset’ which he vigorously denied. The *Spectator* on 18 April 1829 reported that Partridge killed the boy by cutting his throat and then left him dead in a field: ‘As the prisoner had that morning left the place, suspicion attached to him, he was apprehended and in his pocket was found a clasp‐knife stained with blood: there was also blood upon his clothes. The prisoner soon after confessed that he had murdered the boy.’^80^ At his subsequent trial he was found guilty and sentenced to death. Before he hanged however he confessed in prison to his father that he had committed a previous child‐murder. Jonas Ansell aged seven (brother of George Ansell, technically his second victim), had been knifed by Partridge in August 1827. His defence was that he ‘committed the crime at the instigation of an abandoned woman with whom he had co‐habited and who suspected that the child had been a witness to some part of their criminal activity’. It also transpired that he had ‘been repeatedly connected to criminality with three girls’ and been led into the ‘path of mortal sin’. There was little sympathy for a double child murderer who killed for ‘worthless women’. The ‘multitude’ assembled to witness his execution did so, however, according to the *Bury and Norwich Post*, ‘with propriety, so as to refrain from the expression either of sympathy or execration’. Yet, the most shocking fact to emerge did so at the post‐execution event:

The body was cut down after hanging an hour, and conveyed to the Shire Hall, when the public were admitted to pass through, the corpse being laid upon the table with the integuments removed, so as to exhibit the muscles of the thorax. In the evening it was conveyed in the pursuance of a special warrant from the Judge to the Suffolk hospital where it will be dissected, and preparations of various parts, will we believe be preserved.

Earnestly do we wish that this dreadful exhibition may be productive of the salutatory effects upon the minds of both sexes; but it is a melancholy discouragement to the hope of such benefit that George Partridge, with the guilt of one murder already on his conscience, *was actually a spectator at the execution of Corder!* [sic]^81^

All the elements of managing a post‐execution event that this article has highlighted so far are elaborated here. Partridge was cut open at the Shire Hall in Bury St Edmunds because it was still a commodious public space to check the prisoner was truly dead by removing his vital organs and then displaying the corpse for the multitude assembled in 1831. They walked in an orderly fashion down the sides of the dissection table from noon to dusk. In 12 hours around 10,000 people were processed through a public space that was *socially produced* (exactly as Le Febvre thought) and it became a *counter‐site* of punishment (resembling Foucault's speculations) at a rate of not less than 833 spectators an hour or 19 persons a minute. The crowd evidently wanted to see the body cut open because this was a heinous crime. What alarmed many present was the fact that Partridge had been an audience member with the tens of thousands that went to Corder's execution. And as was made clear in subsequent reporting, he had followed his body post‐execution too. Partridge did so knowing he had already covered up one murder and would go on to kill another child soon after. Evidently copycat killing was disturbing to the medico‐legal authorities responsible for deterrence in Suffolk. In many respects however this was not out of the ordinary for this area, for the *Ipswich Journal* had likewise reported on a murderess named Ann Arnold who killed her bastard child and was convicted at the Bury Assizes on 3 April 1813. She too was hanged, cut down and ‘in the afternoon her body was dissected in the Shire Hall [at Bury St Edmunds]’. Again the local newspapers reported that: ‘we were sorry to observe crowds of women and children going thither to view so disgusting a sight’.^82^ The danger of impressionable minds remained a timely problem at the event management of post‐execution rites however well‐planned. We complete this punishment sight‐seeing therefore by returning to Yorkshire where at Leeds the authorities tried to reconcile huge crowds with more controlled event management under the Murder Act.

Samuel Hey (1736–1819) was a renowned surgeon based at Leeds Infirmary where he often received condemned bodies executed at York Castle. If a murder had been a high‐profile event in the community he also saw it as an opportunity to raise revenue for the voluntary hospital where he worked. He thus adopted the standard punishment choreography that was used at Shire Halls across the Midlands. And he did so because he had to accommodate spectators of up to ‘20,000 people at a time’.^83^ His work pattern is symbolic therefore of many *heterotopias of dissection* in provincial life as towns and cities grew outside of London over the course of the Murder Act. Hey hence generally staged his criminal dissections over three consecutive days. On day one, those assembled at the scaffold travelled from York gallows to Leeds Infirmary to see the executed body laid out for their inspection: indicative of the agency local people physically expressed to be involved in the Northern counties, reflecting Midlands’ trends too. Following the execution of Mary Bateman for murder in March 1809, for instance, Hey noted that he charged ‘3d per person’ from ‘24,000 spectators’ after her arrival from the gallows. This was an enormous number of people to event manage at the morgue room set aside for the initial inspection. He cut the body open, checked the major organs had expired, and left it to public view.

On day two, the public door was then closed. Medical men and their apprentices were admitted by a timed‐ticket entry system, in exactly the way that Foucault postulated would happen in a ‘counter‐site’ of punishment. This time Hey charged ‘10s 6d’ each and he also sold tickets that he charged ‘£100 each’ to professional men of Leeds who paid a premium price to be present when the body was extensively cut. Their wives were not excluded either. They were admitted at nightfall on day two but were only permitted to see ‘special lectures for ladies upon the eye’. This was considered seemly and a fitting spectacle for educated female sensibilities. Thus, just as White has stressed the diverse social spectrum of people assembled at executions, here we can observe an equivalent situation at a post‐execution event too.^84^ Overall, Mary Bateman's criminal dissection over three days garnered a profitable ‘£80 14s 0d’ for Leeds Infirmary. Helpfully, the court recorder commented on what he thought attracted ordinary people on day one to the initial opening of the criminal corpse to public view: ‘There was something ‘chilling’, he wrote, ‘gruesome, but ‘fascinating’ about ‘seeing a body filled with guilt’. It was this experiential engagement that held sway in the popular imagination of the dissection theatre, and its dramatic tragedies, of their time.

## V

The *General Advertiser* on 19 September 1752 reminded its readership that: ‘Death is a Portion of every Individual’.^85^ Being in close proximity to someone's imminent demise whether from natural or violent causes: ‘We must surely look upon ourselves as only Tenants at Will, and liable to be dispossessed of our earthly Tabernacle at a Moment's warning’. Nobody could escape the mortal fact that, ‘the Crimson fluid which distributed Health, is impregnated with the seeds of Death’. At a domestic death‐bed scene, on the gallows, or in the dissection room, everyone saw in dramatic terms how: ‘the Partition that separates Time from Eternity is nothing more than the Breath of our Nostrils; and the transition may be made in the least Particle of Time’. Displaying the criminal corpse by medico‐legal officials delineated life from death. Yet, its event management was no easy task, remaining unpredictable inside public premises in which to view the *dangerous dead*. Thus ‘justice was remade from the margins’ outside the Metropolis in English provincial life from 1752 to 1832.^86^

It follows that the post‐execution penalties of the Murder Act have been described in general terms but not researched enough in histories of law and order in England, compared to Europe.^87^ This article has therefore presented new archival evidence detailing criminal dissections (as in Tables 1 and 2) framed by punishment perspectives based on their social geographies and event management (as in Figure 1). Together these have wide application in crime historiography, offering new explanations of potential longevity for studies of the capital statute. Self‐evidently, the agency and people‐flow of the crowds that attended criminal dissections was multifaceted.^88^ It is therefore inaccurate for crime histories featuring criminal dissections to continue to assert that: ‘The seeing was limited to a select audience, whose viewing was not supposed to be voyeuristic but was intended rather to promote the progress of knowledge.’^89^ If it had been there would not have been so much discussion in contemporary newspapers and pamphlet literature concerning the scale of the audiences attending criminal dissections and the threat they posed to public order. The historical lens in this article has hence profiled the obvious but misunderstood practicalities of event‐managing a spectacle of law and order that required penal surgeons to process huge visitor numbers after the scaffold, and for which a high degree of discretionary justice was continually necessary. Those officials repeatedly exploited their position to re‐timetable post‐mortem ‘harm’ to serve multiple agendas that aligned with Enlightenment sensibilities: remaking law and order, crime and justice, medical and scientific knowledge, in the context of refashioning central‐local relations. Facets that extend recent work published on the cultural meaning of the criminal corpse in histories of crime and punishment across European society.^90^

It is also apparent that the private nature of anatomy has been exaggerated in standard historical accounts; its public, multi‐disciplinary, application has often been misconstrued. Dissection should be seen in more‐than‐anatomical‐terms in the broader Enlightenment intellectual climate of changing medico‐scientific mentalities and their punishment application in England. Thus when for example Joshua Slade was hanged at Huntingdon on 1 September 1827 for the ‘foul murder of the Revd, Joshua Waterhouse, late rector of Little Stukeley’, publicity surrounding his case stressed that: ‘the surgeon *acted wisely* in making the dissection *public*’.^91^ In an era when audiences could pay extra to buy a ticket to sit on the actual stage with actors at a playhouse, dissections had picked up on this theatrical trend.^92^ Getting up close to the action, almost touching the main character, and above all being physically involved in the storyline, could be appropriated to serve the spectacle of law and order too.

Gilberto Perez has thus elaborated on the attraction of immersive theatre for audiences, intimating why the genre appears to have historical longevity in European culture: ‘*Suspension* would be an apt word … each image threatens to disclose a monstrous withheld truth, or discharge an act of violence forever hinted but always supressed – heat‐lightening without thunder’.^93^ The author of the punishment drama was the murderer that had committed homicide and been found guilty at the Assize. Yet, the director of the punishment script kept changing hands: from judge to hangman, from penal surgeon to medical student or lay spectators, and all within a polyvalence timeframe and multimodal concepts of the physical remains of the criminal corpse, each contributing to a ‘cultural compost’ of death, dying, and disposal.^94^ Obtaining closure under these complex circumstances involved a lot of cross‐cutting in the presentation of the dramatic narrative of post‐execution punishment. And therefore the *social production* of the penal space (Lefebvre's cultural framework) as a *counter‐site* of retribution (Foucault's *heterotopias of dissection*) had to align with architectural features that folded ‘together *place, performance and public*’ (Wiles’ play on the theatre of tragedy). This cultural trinity involved event‐managing different types of spectacles because punishment steps happened *within* scenes (strangled and/or hanged by the executioner), as well as *between* scenes (washed, cut open, displayed, engaging with different dissections ticket‐timed over several days). Continually, each criminal corpse's visual appeal lying on a dissection table was the equivalent of a camera close‐up where physical stillness can still communicate to the audience powerfully. For, absence was always as dramatic as presence in the 692 criminal dissections investigated for this article. Above all, it was the condemned centre‐stage looking lifeless that conveyed closure in the silence of its penitent soliloquy. Material display by medico‐legal officials was all about trying to meet an audience's sense of performance closure or risk a public order situation getting out of control. Yet, in the visual value of the criminal corpse displayed and dramatized for deterrence purposes, it was body‐time running‐out at dissections that did the parallel‐editing in the tragedies scripted by the Murder Act.

## Footnotes

1 See, admirably, V. A. C. Gatrell, *The Hanging Tree: Execution and the English People, 1770–1868* (Oxford, 1996).

2 M. White, ‘Lex Talionis’, *Encyclopaedia of Law and Economics* (New York, 2014), pp. 1–2.

3 Selectively, P. Linebaugh, *The London Hanged: Crime and Civil Society in the Eighteenth Century* (London, 1996); R. Shoemaker, *The London Mob: Violence and Disorder in Eighteenth Century London* (London, 2004); D. Hay et al., *Albion's Fatal Tree: Crime and Society in Eighteenth‐Century England* (London, 2011); P. J. R. King, *Crime and Law in England, 1750–1840: Remaking Justice from the Margins* (Oxford, 2010).

4 The gibbet tended to be reserved for traitors, highwayman and pirates, as well as those who robbed the Royal Mail or threatened the policing infrastructure of the early modern state; hanging in chains was also extended to 20% of murderers of heinous acts of violence like rape or incest that killed the victim. See S. Tarlow, *The Golden and Ghoulish Age of the Gibbet in Britain* (Basingstoke, 2016).

5 Published collectively in, *Palgrave Historical Studies in the Criminal Corpse and its Afterlife*, <http://www.springer.com/series/14694> [accessed 6 Oct. 2017]. I am grateful to the anonymous referees who gave such valuable feedback and constructive criticism to an earlier version of this article. This research has been funded by a Major Wellcome Trust Programme Grant (2012–17), WT095904AIA ‘*Harnessing the Power of the Criminal Corpse*’ at the University of Leicester.

6 Under the Anatomy Act (1834) those who died in welfare institutions and became ‘unclaimed’ were sent for dissection. See E. T. Hurren, *Dying for Victorian Medicine: English Anatomy and its Trade in the Dead Poor, c. 1832 to 1929* (Basingstoke, 2011).

7 C. P. Graves, ‘From archaeology of iconoclasm to an anthropology of the body: images, punishment and personhood in England, c. 1500–1660’, *Current Anthropology*, 49/1 (2008), pp. 35–57, at p. 35.

8 A point made throughout, E. T. Hurren, *Dissecting the Criminal Corpse: Staging Post‐Execution Punishment in Early Modern England* (Basingstoke, 2016), ch. 5.

9 See ibid., ch. 3.

10 Reviewed in A. Vanhaelen and J. P. Ward (eds), *Making Public Space in Early Modern Europe: Geography, Performance*, *Privacy* (New York and Abingdon, 2013), quotes at pp. 4, 9 and 17.

11 Ibid., p. 17, and D. Wiles, *A Short History of Western Space* (Cambridge, 2013), p. 12.

12 M. White, ‘ “Rogues of the meaner sort?”: Old Bailey executions and the crowd in the early nineteenth century’, *London Journal*, 33/2 (2008), pp. 135–53, quotes at p. 148.

13 King, *Crime and Law*, p. 1.

14 See, notably, R. Richardson, *Death, Dissection and the Destitute* (Chicago, 1987); H. McDonald, *Human Remains: Dissection and its Histories* (New Haven, CT, 2005).

15 Exemplified in A. Cunningham, *The Anatomical Renaissance: The Resurrection of the Anatomical Projects of the Ancients*, (Aldershot, 1997); idem, *Anatomy, Anatomis'd: An Experimental Discipline in Enlightenment Europe* (Aldershot, 2010).

16 Y. Hamilakis, M. Pluciennik and S. Tarlow, *Thinking Through the Body: Archaeologies of Corporeality* (New York and London, 2002), p. 89.

17 See S. Tarlow, *Ritual, Belief and the Dead in Early Modern Britain and Ireland* (Cambridge, 2013).

18 S. Tarlow et al., ‘The body in the age of knowledge’, in J. Robb and O. T. Harris (eds), *The Body in History: Europe from the Palaeolithic to the Future* (Cambridge, 2013), pp. 164–88, quote at p. 181.

19 Ibid., p. 191.

20 Graves, ‘From archaeology’, p. 41.

21 Vanhaelan and Ward, *Making Public Space*, p. 4.

22 M. Foucault, ‘Des espaces autres’, *Architecture, Mouvement, Continuité*,* *5 (1984), pp. 46–9, <http://www.foucault.info/documents/heterotopia/foucault.heterotopia.en.html> [accessed 10 April 2017]; idem (trans. Jay Miskowiec), ‘Of Other Spaces’, *Diacritics*, 16/1 (1986), pp. 22–7; idem, ‘Different spaces’, in R. Hurley and J. D. Faubion (eds and trans.), *Aesthetics, Method, and Epistemology: Essential Works of Foucault, 1954–1984*, II (New York, 1998), pp. 175–85; P. Johnson, ‘Unravelling Foucault's “different spaces” ’, *History of the Human Sciences*, 19/4 (2006), pp. 75–90.

23 Wiles, *Western Space*, p. 5.

24 From the perspective of social geographers, see, notably, K. Hetherington, *The Badlands of Modernity: Heterotopia and Social Ordering* (London, 1997).

25 See <http://www.heterotopiastudies.com/whats-it-about/> [accessed 19 April 2017].

26 Highlighted in E. T. Hurren, ‘The dangerous dead: dissecting the criminal corpse’, *Lancet*, 382/9889 (2013), pp. 302–3.

27 Wiles, *Western Space*, quotes at pp. 1–2.

28 Ibid., p. 1.

29 Sometimes new evidence did come to light to exonerate the crime of murder after a trial for homicide was finished. Under the capital code, judges thus had the discretion to issue a full pardon, or pass a lesser sentence like transportation, provided they did so before the Assizes closed and they departed officially from the area of the court circuit.

30 An important observation first made by S. Chaplin, ‘John Hunter and the Museum Oeconomy, 1750–1800’, unpublished PhD Thesis, King's College London (2009) and recently substantiated by Hurren, *Dissecting the Criminal Corpse*, pp. 120–54.

31 The 102 cases examined at The National Archives [hereafter TNA] include: E389–243 to E389–254; 90–147 to 90–169 (1752 to 1832). These are tested against surviving Assize records in England. Representative cases were cross‐matched to newspaper accounts and penal surgeons’ profiles held in county record offices. This is the most extensive attempt that has been made to correlate homicide rates with criminal dissections. I am grateful to Dr Richard Ward for acting as a research assistant and compiling the statistical figures on behalf of the criminal corpse research team at the University of Leicester.

32 Further details can be read in, Hurren, *Dissecting the Criminal Corpse*, ch. 3.

33 Context outlined (for instance) in T. Nunnely, *Introductory Lecture delivered at the Leeds School of Medicine at the Opening of the 22nd Session 4 October 1852* (London, 1852), pp. 1–27, quote at p. 27, in which he extolls the dissection work of the recent past, as: ‘I think I have observed . . . there has been a growing disposition to rely on well‐illustrated and beautifully got up works upon anatomy which have of late years issued from the press, rather than undergo the trouble [Murder Act] and drudgery of dissection [Anatomy Act]. Against this let me warn you. Illustrations and plates are most valuable assistance and *capital* refreshers; but they cannot *teach* anatomist . . . To become an anatomist there is no short cut – no Royal road to proficiency; the only way is to cut open its votaries is that by which so many of our predecessors have ascended the rugged path of eminence – careful and repeated dissection’.

34 O. Davies and F. Matteoni, ‘ “A virtue beyond all medicine”: the hanged man's hand, gallows tradition and healing in eighteenth‐ and nineteenth‐century England’, *Social History of Medicine*, 4 (2015), pp. 10–28.

35 TNA, Sherriff's Cravings, Chester (1812) and *The Times*, 21 Aug. 1812 and 29 April 1813.

36 See J. Sawday, *The Body Emblazoned: Dissection and the Human Body in Renaissance Culture* (New York and London, 1996) on the fearful prospect of seeing this too.

37
*Times*, 29 April 1813, reporting was detailed.

38 Ibid.

39
*Times*, 29 April 1813, carried a detailed account.

40 A procedure highlighted in, Hurren, ‘The dangerous dead’, pp. 302–3.

41 See n. 14 above, where historiography has ignored event management.

42 Nottingham Record Office [hereafter NRO], Chamberlain's Accounts, entry dated 10 April 1775, expenses being reclaimed for the costs of William Voce's execution and dissection.

43 J. F. Sutton, *The Date‐book of Remarkable and Memorable Events Connected with Nottingham* (Nottingham, 1852), p. 109, entry for 1775.

44 Ibid., p. 239, entry for 1799.

45 Sutton, *The Date‐book*, p. 316, entry for 1815.

46 Anon., *The Edinburgh Gazetteer, or compendious geographical dictionary, forming a complete geography of political, statistical and commercial landscape, in six volumes* (Edinburgh, 1829), p. 773, Wakefield dispensary.

47
*Leeds Mercury*, 22 March 1817, carried an extensive report of the trial on its front pages.

48 See *The Annual Register*, LIV (London, 1812), pp. 92–3.

49 British Library newspaper archive, *Strabane Morning Post*, 21 June 1812, ‘Winchester Assize: murder of Elizabeth Hill, Shalfleet, and execution of John James at Winchester’, p. 1.

50 See A. Clarke, *A Sermon Preached in the Cathedral Church of Winchester, before the Governors of the County‐Hospital for the Sick and lame & so on, and at the opening of the said Hospital on St. Luke's Day October 18 1736 etc*. (London, 1737), p. 1 and appendix.

51 See Rev. Dr Milner, *A Short View of the History and Antiquities of Winchester, with a brief account of the Seats of the Neighbouring Nobility, Gentry & so on, 5th edition* (Winchester, 1812), pp. 22, 43, 44.

52 Gatrell, *Hanging Tree*, convincingly makes this point.

53 R. Poole, ‘Give us our eleven days!’: calendar reform in eighteenth‐century England’, *Past and Present*, 149 (1995), pp. 93–139, describes this general time‐keeping issue.

54 P. Glennie and N. Shrift, *Shaping the Day: A History of Timekeeping in England and Wales, 1300–1800* (Oxford, 2009), intro.: sect. 1.3. Trends confirmed in S. A. King and G. F. Timmins, *Making Sense of the Industrial Revolution: English Economy and Society 1700–1850* (Manchester, 2001).

55 V. Browne, *Feminism, Time and Nonlinear History* (Basingstoke, 2014).

56 O. Davies and F. Matteoni, *Executing Magic in the Modern Era* (Basingstoke, 2017).

57 S. McCorrestine, *Spectres of the Self: Thinking about Ghosts and Ghost‐seeing in England, 1750–1920* (Cambridge, 2010).

58 A. Gingrich, E. Ochs and A. Swedland, ‘Repertories of timekeeping in anthropology’, *Current Anthropology*, 43/1–3 (2002), pp. 1–2, quote at p. 1.

59 It is noteworthy that the recent reburial of King Richard III sparked considerable media commentary about general access to the view of the body. Whilst the historical context here is different, its experiential elements in terms of spectatorship, notoriety and public curiosity do appear to have an intriguing cultural longevity.

60 See P. Spierenburg, *The Spectacle of Suffering: Executions and the Evolution of Repression: From a Pre‐Industrial Metropolis to European Experience* (Cambridge, 2009).

61 See R. Porter, *English Society in the Eighteenth Century* (London, 1990).

62 ‘Execution of A. and M. McKeand for the murder at Winton’, *The Observer*, 27 Aug. 1826, pp. 4–5.

63 On his penal career, see, TNA, *BPP*, (1812–3), V, *Report of the Commissioners on the State Lancaster Prison and the Treatment of Prisoners Therein*, p. 34, and later printed as *State of Lancaster Gaol* (London, 1812), entry 3 July 1812, pp. 897–8.

64 ‘Execution of the Keands’, *The Manchester Guardian*, 26 Aug. 1826, pp. 1–2.

65 ‘Murder at Winton: execution of the Keands’, *The Age*, 26 Aug. 1826, p. 542.

66
*Caledonian Mercury*, 31 Jan. 1829, p. 3. I am grateful to Dr Rachel Bennett for sharing this source that features in her forthcoming book (2018) on the Murder Act in Scotland.

67
*The Morning Post* 14 Aug. 1828, carried extensive reports of the execution and post‐execution events and the reaction of the early modern crowd over several days.

68 Ibid.

69 Aristotle, *Poetics*, VI, 1449b, 2–3, <http://www.perseus.tufts.edu/hopper/text?doc=Perseus%3Atext%3A1999.01.0056%3Asection%3D1449b> [accessed 11 April 2014].

70 ‘Thomas Grundy murder’, *Leicester and Nottingham Journal*, 29 March 1788. Grundy was convicted at the Lent Assizes on 22 March 1788.

71 NRO, BB34.8, ‘Gallows Rememberancer for 1763’, p. 25.

72 Dorset Record Office, NG/PR1, Prison Admission and Discharge Registers, 1782–1901, ‘Case of John Anderson Constable arrest of Elizabeth, otherwise known as Betty Marsh’; D/SEN/3/7/8, and execution 22 June 1792 rites. Confirmed by a bond between the two main surgeons of Dorchester, John Coombs and Philip Coombs; D/SEN/3/7/10–11.

73 Editorial, ‘Murders in Cornwall’, *A Journal of The Universal Magazine of Knowledge and Pleasure, Provincial Occurrences* (December, 1814), pp. 517–18, where it was alleged that William Burns may have also killed a man in Dublin. See likewise R. M. Barton, *Life in Cornwall in the Early Nineteenth Century: Being Extracts from the West Briton Newspaper in the Quarter Century from 1810–1835* (Truro, 1970).

74 TNA, T90/170, Burns (Cornwall, 1814) ref. 7306891, expenses claimed back by Joseph Edwards, Undersheriff.

75 Leicestershire Record Office, Leicester Infirmary Minute Books, 20 Aug. 1816, and further such concerns about the need for a different sort of public staging discussed on 8 April 1817, 5 Aug. 1817 and 10 Aug. 1817 meetings.

76 Ibid., 24 and 29 March 1825.

77 Hurren, *Dying for Victorian Medicine*, chs 3 and 6, provide a detailed discussion of commissioned dissection tables. At Manchester Infirmary, anatomists bought John Hunter's rotating table when it came up for auction after his death.

78 See also White, ‘Rogues’, pp. 135–53, where strong emotional attachments to punishment rites created community memories.

79 See n. 11 above.

80 ‘Lent Assizes – Murder‐ George Partridge was tried at Bury St Edmunds’, *The Spectator*, 18 April 1829, p. 5.

81 ‘The Murderer Partridge’, *Bury and Norwich Post*, 15 April 1829, pp. 4–5.

82 ‘Ann Arnold – murder of her bastard child’, *Ipswich Journal*, 3 April 1813, p. 2.

83 Leeds University, Special Collections, MS 504/1/3, Mr William Hey – Senior Surgeon to Leeds General Infirmary.

84 White, ‘Rogues’, pp. 135–53.

85 Editorial, *General Advertiser*, 16 Sept. 1752, pp. 1–2.

86 King, *Crime and Law*, p. 1.

87 See also S. Poole, ‘For the benefit of example: crime scene executions in England 1720–1830’, in R. Ward (ed.), *A Global History of Execution and the Criminal Corpse* (Basingstoke, 2015), pp. 71–101.

88 See also: R. B. Shoemaker, ‘Streets of shame? The crowd and public punishments in London, 1700–1820’, in S. Devereaux and P. Griffiths (eds), *Penal Practice and Culture, 1500–1900: Punishing the English* (Basingstoke, 2004), pp. 232–57; G. F. Rudé, *The Crowd in History: A Study of Popular Disturbances in France and England, 1730–1848* (London, 2005); I. Munro, *The Figure of the Crowd in Early Modern London: The City and its Double* (Basingstoke, 2005); J. Walter, ‘Crown and crowd: popular culture and popular protest in early modern England’, in J. Walter (ed.), *Crowds and Popular Protest in Early Modern England* (Manchester, 2006), pp. 14–26; F. Burwick, *Playing to the Crowd: London Popular Theatre, 1780–1830* (Basingstoke, 2011).

89 R. McGowan, ‘Making examples and the crisis of punishment in mid‐eighteenth‐century England’, in D. Lemmings, *The British and their Laws in the Eighteenth Century* (Woodbridge, 2005), pp. 182–203, quote at p. 203.

90 Notably, Spierenburg, *The Spectacle of Suffering*.

91 Huntingdon Record Office, 2913/2/6, Dickinson Collection, Huntingdon village notes 1827, 4230A, Joshua Slade execution broadside 1 Sept. 1827, MC/12/1–18, Report on the Reverend Waterhouse Murder 1827.

92 Tickets to sit on the stage were often sold to be part of a ‘frame story’, a device to make light of tragedy. Mary Shelley's use of it in *Frankenstein* for instance explores the power of medicine to resuscitate the dead, in D. Herman, M. Jahn and M. L. Ryan (eds), *Encyclopaedia of Narrative Theory* (London, 2013), p. 134.

93 G. Perez, *The Material Ghost: Films and their Medium* (Baltimore, MD, 1998), p. 368.

94 Apt phrasing by A. Nicholson, *The Gentry: Stories of the English* (London, 2011).

